# Commentaries on Some Doctrines of a Dangerous Tendency in Medicine, and on the General Principles of Safe Practice

**Published:** 1843-04-01

**Authors:** 


### COMMENTARIES ON SOME DOCTRINES OF A DANGEROUS TENDENCY IN MEDICINE, AND ON THE GENERAL PRINCIPLES OF SAFE PRACTICE. By Sir *Alexander Crichton*, M.D., F.R.S., &c. &c. 8vo. pp. 283. Churchill, London, 1842.

THIS is one of those books that are incapable of analysis, and for this reason, that these Commentaries are so many critiques on theories and practices—doctrines and dogmas—men and things. When we reflect that these Commentaries were written when the author was close bordering on the 80th year of his age—that the style is vigorous and perspicuous—that the information, physiological, pathological and therapeutical, comes down to the very instant of publication, we are absolutely astonished at the apparently unimpaired strength of mind and general intellectual powers of this venerable octogenarian!! He must be a descendant of the "ADMIRABLE CRICHTON!"

The work consists of three Commentaries, and a large Appendix of Cases and Reports from the venereal wards of the Hamburgh Hospital, illustrating the non-mercurial treatment of syphilis. The first Commentary is on "the functions of the heart and arteries in health and disease," and opens up the long disputed question whether arteries are muscular or elastic, or both. In this part, the doctrines of physiologists, from Harvey and Haller to Young and Philip, are critically examined and commented on, and the doctrines and experiments of the *latter* very roughly handled. Sir A. comes to nearly the same conclusion as Dr. Young did, that the circulation depends on the muscular force of the heart and the ELASTIC contraction of the arteries on the blood injected into them—a doctrine so beautifully illustrated and proved by the late Dr. Parry, of Bath, to whose experiments our author does not allude.

In an appendix to the preceding commentary, Sir A. makes some useful remarks on "exercise and inaction," though nine-tenths of the world are unable or unwilling to apply them to themselves—because they are not, in fact, masters of their own actions. After shewing the errors and bad consequences of violent exercise succeeded by inaction and indulgence, Sir A. observes:—

"A good rule for the employment of exercise, with the view of prolonging life, consists in taking it, not once, nor even twice, but several times a day; for by so doing, the force of the circulation is sustained, and organic structure safely increased, on the principle on which all parts are nourished.

"Bodily inaction ought not to be persisted in for upwards of four hours consecutively, if perfect health is the object, except during sleep. And for people in health this tempting state of repose ought not in general to be prolonged above seven hours. For the last forty years of my life, I have made it a rule not to remain longer quiescent, during the day, than three hours at a time; and I attribute the good health I enjoy at my advanced age, chiefly to this cause; for I must confess that I scarcely owe anything to dietetic rules, having never been a rigid observer of them." 39.

The second Commentary is on the doctrines and practices relating to fevers. In this, our author comments on various systems and their originators—from Clarke and John Brown, down to Broussais and the present practitioners. The doctrine of topical inflammation as the cause of adynamic fevers, viz. those of Clutterbuck, Broussais and all that class, are severely criticised by our author, who seems to labour under a hœmaphobia; or dread of blood-letting. Sir A. inclines to the doctrine that the matter of contagion acts primarily on the blood, vitiating its composition, and afterwards on the brain and nervous system, thus deranging all the various functions of assimilation, secretion, digestion, &c. He inclines to the doctrine of Liebig, viz. that "if the exciting agent of chemical metamorphosis be a compound body, which, in the act of decomposition, it will re-produce itself *ad infinitum*, provided the compound body on which it acts contains elements fitted for such an end."

We shall not, however, follow our author through all the mazes of theory and speculation, from which, we regret to say but little profit can be drawn. Come we then to the treatment of fever of a typhoid character. Our author observes, that it has always been a primary object to allay the perturbation in the circulation, though the means have varied with the theoretical views of the practitioner. The analogy between a continued and intermittent fever, is perfect at the beginning, but ceases after the first day or two. Still he thinks the *causes* of continued and intermittent fevers differ more in the degree of force or dose than in kind.

"The process of nature, by which a paroxysm of ague terminates favourably, is by profuse perspiration; that is, by the loss of a large quantity of circulating fluids. But this fluid is not blood. It consists of a portion of the liquor sanguinis, which has lost all vitality, and is loaded with chemical compounds that have been formed during the cold and hot stages of the paroxysm; during which the excretions by the urine, by the skin, and by the lungs, have been suppressed." 124.

Sir A. avers that he has rarely seen any success attendant on the attempt to imitate Nature in the manner above described; viz. by sudorifics.

"The process of unassisted nature in curing the long protracted paroxysm of a continued fever is conducted on the same general principle as that which she observes in the paroxysm of an ague; that is, she reduces the quantity of circulating fluids until she brings about an equilibrium between them and the weakened forces which are to propel them, and when this happens, a critical sweat now and then occurs. But in agues, the energy of the brain being less affected than in typhus, the heart and arteries exert greater influence, and consequently, the canals through which perspirable fluids pass, are sooner forced open by the quickened action of the heart, and continued pressure of blood a tergo." 129.

Our author's experience of blood-letting in typhoid fevers amounts to this :—"that it is a hazardous practice if adopted as a general rule; and that in any case, even of suspected inflammation or threatening congestions, it ought seldom if ever to exceed two ounces at a time."

In synochus, with full and quick pulse, with delirium or severe headache, flushed countenance, intense heat, and general pain, Sir A. would not object to the abstraction of eight or ten ounces of blood from the arm; but would prefer the application of six leeches to the neck, with warm poultices: afterwards cold applications to the head, and alterative doses of calomel. Sir A. has never seen a single case of acute inflammation of the peritoneum, liver, or intestines, "even in the first stage of typhus." We certainly cannot completely corroborate this observation by our own experience; for if every criterion of active inflammation was not entirely fallacious, we have very frequently seen the complication of low fever and inflammation, especially of the lungs, in the Winter season. Our author thinks that, ever since the days of Cullen, emetics in the early, perhaps in all stages of typhus, have been too much in disuse. A favourite emetic of Sir

A. is a scruple of ipecacuanha with one drachm of oxymel of squills. When petechiæ or vibices shew the impoverished state of the blood, the mineral acids are indicated. Warm baths are considered as doubtful remedies. They have sometimes more than answered his expectations—at others aggravated the complaint. Sir A. thinks that twenty minutes in the bath is sufficient.

"Upon taking him out of the bath he ought to be instantly conveyed to his bed, from which the sheets have been previously removed. He may either lie between two fine light woollen blankets, or in fine flannel. The moisture on his skin is then soon converted into vapour, which promotes the perspiration. The patient ought not to be dried when he comes out of the bath." 139.

Sir A. recommends a trial of the belladonna practice, as employed by Dr. Graves of Dublin, and noticed in this Journal some time ago. The author, however, has no experience of the remedy. But the febrifuge which has found most favour in his sight, is the cold affusion or bath of Wright, Currie, and other eminent physicians. Sir A. prefers immersion to aspersion, as in the *latter* operation the patient must be kept upright and is apt to faint, in which state it is dangerous to proceed.

"The immersion may be employed at any period of typhus, provided the skin be hot and dry, and the pulse moderately full; but it is chiefly useful in the early stages of the disorder, before the patient is too much exhausted, and probably before local inflammations happen." 150.

The third Commentary is on the mysterious subject of INSANITY. Sir A. justly observes that there is not a single mental manifestation belonging exclusively to insanity. Every individual phenomenon in this disease, may be, and often is, produced by other disorders, as delirium, fevers, metastases, hysteria, &c. &c. The following passage would seem to clear the way to that precious desideratum—A DEFINITION OF INSANITY.

"In every case of disordered intellect which does not fall within the true character of insanity, we discover decisive symptoms of other disorders, which either precede or accompany the aberration of reason, and which account for the mental phenomena; whereas, in true insanity, let its variety be what it may, even acute mania, in which the whole faculties of the mind are in a state of great confusion, there are no corporeal symptoms to which the psychological phenomena can be referred; for, although it cannot be denied that in cases either of acute mania or of maniacal melancholy some symptoms of bodily disorder may at times be discovered, yet these are so disproportionate to the mental affection, and are so uncertain and variable in their appearance, that they cannot be considered as essentially connected with the malady." 160.

Our author admits that certain morbid conditions of the brain, "as tumor, may produce palsy and insanity," but he goes on to say, that "it does not follow that the cerebral affection which produces mania, must, of necessity, occasion palsy." No, but it proves that physical alterations do produce insanity in some cases, and if so, why not in all cases? After shewing the unsatisfactory terms in which definitions of insanity have hitherto been exhibited, Sir A. ventures on his own: voila—

"*Insanity is a disease of the brain, which causes the patient, while awake, to mistake the phantoms and operations of imagination for realities, which, conse-*

*quently, become the motives of his discourse and actions, while at the time there is an absence of any bodily disorder that can account for the phenomena.*" 165.

Now as brevity is the most valuable quality of a definition, why not say explicitly that "INSANITY IS A WAKING DREAM," for this is the sum and substance of the above definition? How far it will stand the test of the jury-box we do not pretend to say; but we confess that we should mount that rostrum with some trepidation, were we only armed with Sir Alexander's shield. "MANIA IS A DISEASE OF THE BRAIN," says our author; but he does not clearly impart to us what that disease is. The preliminary symptoms generally evince "*cerebral excitement;*" but not always, and therefore cannot be taken as the pathognomic phenomenon.

"But there is one preliminary symptom of insanity, in general, which is common to all the foregoing classes, namely, an almost complete change of moral character." 167.

Our author repudiates the doctrine of insanity being a disease of the mind, if by mind we mean the soul, or the "immaterial agent which directs our reason or moral feelings."

"The brain is the only viscus of the human body which performs two functions that are essentially distinct from each other. There is, in fact, not a shadow of resemblance between them; and this justifies the conclusion that there are two distinct agencies operating on the brain, to which the two classes of phenomena are to be referred. One of these agents is the living principle which is common to all the organs of the body, by the laws of which, its animal functions are performed and maintained; such as its nutrition or renovation, and the forming a peculiar stimulant, which is conveyed by the nerves to every part to which they are distributed. The other is that mysterious and spiritual agent, to which conscience, and reason, and indeed all the intellectual faculties, must be referred." 169.

Our author justly considers the anatomical researches of Foville and Delaye as the very best that have ever been made in respect to the condition of the brain in insanity. These gentlemen always took care to have a sound brain on the table while dissecting the brain of a maniac. In acute cases of insanity, the cineritious part of the brain was discovered by these gentlemen to be preternaturally red and congested. In early stages of acute insanity, no adhesions of the membranes to the cortical substance were discovered; but, in chronic cases these adhesions were very common. Dr. Carpenter has come to very similar conclusions.

### "REFLECTIONS ON REMEDIAL TREATMENT."

Several eminent physicians, especially Rush, Haslam and others, have considered acute mania as closely allied to inflammation, and have vouched for the great benefit derived from blood-letting in the early stages. Sir A. demurs to these statements; but as he never had the charge of a lunatic hospital or asylum, he offers his opinions with some degree of diffidence. He strengthens his anti-venesections, however, by many good authorities, as Pinel, Esquirol, and others. Sir A. is convinced that, although the anatomical characters of inflammation are cognizable in the brain after death, yet that they are not the sequences of actual inflammation—in short, that maniacal inflammation is of a specific kind, and not amenable to the same remedies as common phlogosis.

Of all the remedies which our author has employed in the treatment of acute mania, the tartrate of antimony has proved the most efficient, in doses of one-fourth to one-sixth of a grain every four hours. It must be employed uninterruptedly for some weeks, if the paroxysm lasts so long. He has sometimes combined it with camphor, but not at the beginning. In conjunction with this remedy, he puts the patient daily into a tepid half-bath for half an hour. The bowels are to be kept open once or twice a day. In the latter years of his practice Sir A. was in the habit of giving a grain of extract of colchicum—four grains of colocynth—and a quarter of a grain of belladonna at night, as an aperient sedative. The shaven head is recommended to be frequently sponged with cold vinegar and water. When the scalp is hot, and the delirium excessive, he applies some leeches to any part of the head. Blistering the scalp, and affusions of cold water he considers as hazardous. Low diet in the acute stage, and generous fare in the stage of collapse are properly advised.

There are a few pages dedicated to, "THE CRIMES WHICH ARE IMPUTED TO INSANITY," but this small portion of the work needs not any special notice in this Journal.

Some 80 or 90 pages are devoted to a subject that has now nearly lost all its interest—the treatment of syphilis without mercury. There are still some surgeons in this country who try this experiment on others, but very few, we suspect, who would do so on themselves. The reports of Dr. Gunther and Fricke, at the Hamburgh Hospital, in 1828, were in accordance with many other reports of a similar nature in this country—namely, that the treatment of syphilitic diseases was not only more easy without than with mercury; but that fewer cases of secondary symptoms occurred.

> "At the present moment (says Fricke,) in which this is written, (February, 1828,) and after a lapse of a year and a half since the non-mercurial plan of cure began, and after one thousand patients have been subjected to it, the result has been so fortunate, that we have no reason for abandoning it or of recurring to the old practice." 209.

Alas! Eighteen months afford but a very uncertain basis for the substantiation of a new doctrine or practice! Fourteen or fifteen years afterwards, and the non-mercurial practice was scarcely thought of, except in some rare cases where mercury was found to be inadmissible. The new practice, however, was productive of much good. It taught us to use mercury with less prodigality—that very low living tended greatly to suspend the progress of syphilis—and to prove a valuable auxiliary to the moderate and judicious administration of the specific mineral. This is a greater good than has resulted from any new practice that has not been able to fulfil its original pretensions.

We now take leave of our venerable and highly respected author, concluding with the Oriental salutation—"MAY YOU LIVE A THOUSAND YEARS."

